# Interaction of Catechol‐O‐methyltransferase Val^158^Met polymorphism and sex influences association of parietal intrinsic functional connectivity and immediate verbal memory

**DOI:** 10.1002/brb3.1784

**Published:** 2020-08-08

**Authors:** Sichu Wu, Neeraj Upadhyay, Jiaming Lu, Xueyan Jiang, Shumei Li, Zhao Qing, Junxia Wang, Xue Liang, Xin Zhang, Bing Zhang

**Affiliations:** ^1^ Department of Radiology Drum Tower Hospital Medical School of Nanjing University Nanjing China; ^2^ German Center for Neurodegenerative Diseases (DZNE) Bonn Germany

**Keywords:** COMT Val^158^Met, dopamine, functional connectivity, immediate verbal memory, parietal lobule, resting‐state fMRI, sex

## Abstract

**Introduction:**

Sex differences modulate catechol‐O‐methyltransferase (COMT) genotype effect at a synaptic dopamine level, which influences brain function as well as cognitive performance. In this study, we investigated how COMT Val^158^Met polymorphism and sex affect intrinsic functional connectivity and memory.

**Methods:**

Intrinsic functional networks were extracted using independent component analysis of resting‐state functional magnetic resonance imaging data from 186 healthy young COMT‐genotyped participants. The association of these functional networks and memory function was tested to investigate whether the effect of COMT × sex interaction influences the association of intrinsic functional connectivity and memory performance. Quadratic curve fit estimation was used to examine the relationship between functional connectivity and speculative dopamine level among groups.

**Results:**

COMT MM/MV carriers, relative to VV carriers, showed increased functional connectivity in left superior parietal lobule and right inferior frontal gyrus. Further, male MM/MV carriers showed significant higher mean functional connectivity in left inferior parietal lobule relative to male VV carriers and female MM/MV carriers, which was associated with worse immediate verbal recall performance. Additionally, the relationship between inferior parietal lobule functional connectivity and speculative dopamine level among groups fits the quadratic curve.

**Conclusions:**

These findings suggest that the interaction of COMT genotype and sex might regulate synaptic dopaminergic concentrations and influence the association of intrinsic functional connectivity and immediate verbal memory in left inferior parietal lobule.

## Graphical abstract

Synaptic dopaminergic concentration in human brain influences brain function and memory performance, which can be modulated by both COMT Val158Met polymorphism and sex. To investigate how COMT and sex affect intrinsic functional connectivity and memory, we used independent component analysis to examine the influence of COMT genotype × sex interaction on association with functional networks and memory function. We found that male Met carriers, who are hypothesized to have higher dopaminergic concentrations, show higher functional connectivity in left inferior parietal lobule which is related to impaired immediate verbal recall performance, thus providing supporting evidence for the pathophysiology of schizophrenia.

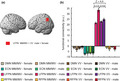

## 1 INTRODUCTION

Catechol‐O‐methyltransferase (COMT) enzyme, which is encoded via the COMT gene and located at 22q11.2 (Grossman et al.,^1992^), is responsible for clearing catecholamine neurotransmitters (e.g., dopamine and norepinephrine) in human brain (Tunbridge, Harrison, & Weinberger, 2006). It is regulated by a single nucleotide polymorphism which leads to a valine (Val) to methionine (Met) substitution at codon 158 (Val^158^Met) (Lachman et al., 1996). This substitution results in a three‐ to fourfold decrease in COMT enzyme activity that leads to increased synaptic dopaminergic concentrations in human brain in Met relative to Val carriers (Lotta, Vidgren, & Tilgmann, 1995). Consistent to this, pharmacological studies that manipulate dopamine concentrations in human brains demonstrate that drugs that inhibit COMT enzyme activity, for example, tolcapone, improved behavioral performance in Val carriers during working memory, verbal memory, or executive function (Apud, Mattay, & Chen, 2007; Farrell, Tunbridge, Braeutigam, & Harrison, 2012; Giakoumaki, Roussos, & Bitsios, 2008). Further, estrogen can also down‐regulate COMT activity and increase dopaminergic concentrations in females relative to males (Jiang, Xie, Ramsden, & Ho, 2003; Xie, Ho, & Ramsden, 1999). It is demonstrated that sex influences dopamine levels and behavior in COMT knockout mice ( Gogos, Morgan, & Luine, 1998) and effect of COMT on behavioral performance such as executive control ( Holtzer et al., 2010), facial recognition ( Kempton, Haldane, & Jogia, 2009), or verbal ability ( O’Hara et al., 2006).

Resting‐state functional magnetic resonance imaging (fMRI) is a very useful method to evaluate interactions between intrinsic brain regions when participants are at rest (Raichle, 2011) and reflect the human brain's functional architecture during cognition as well ( Smith, Fox, & Miller, 2009). Resting‐state fMRI (RS‐fMRI) studies have identified the effect of COMT and COMT × sex interaction on several functional resting‐state networks (RSNs) such as default mode network (DMN), executive control network (ECN), and fronto‐parietal network (FPN) that are highly associated with cognitive functioning (Elton, Smith, Parrish, & Boettiger, 2017; Liu, Song, & Li, 2010; Tang, Li, & Xu, 2019; Tian, Qin, & Liu, 2013; Tunbridge, Farrell, Harrison, & Mackay, 2013). Several behavioral and task‐fMRI studies also provide supporting evidence to demonstrate the relationship between COMT and memory performance (Bertolino, Rubino, & Sambataro, 2006; Egan, Goldberg, & Kolachana, 2001; Frias et al., 2004; Raz, Dahle, Rodrigue, Kennedy, & Land, 2011), as well as COMT × sex interaction effect on cognitive performance (Holtzer et al., 2010; Kempton et al., 2009; O’Hara et al., 2006). However, the question of whether differences in synaptic dopaminergic concentration that arises from COMT × sex interaction effect influences the association between RSNs and memory performance remains unclear. Still, brain structure has also been reported to be influenced by COMT and sex. Some previous studies have revealed COMT × sex difference in gray matter volume in prefrontal regions (Kates, Antshel, & AbdulSabur, 2006; Tian et al., 2013; Xu, Qin, & Li, 2016a), whereas others have found no relationship between COMT genotype and sex on gray matter volume (Barnes, Isohanni, & Barnett, 2012; Zinkstok, Schmitz, & van Amelsvoort, 2006). Therefore, whether COMT × sex interaction influences the gray matter volume in healthy young people still needs to be explored.

In the current study, we investigated the effects of COMT genotype and sex on RSNs and memory functions in healthy young participants. Within the RSNs, we focused on DMN, ECN, and FPN that are highly associated with cognitive functions and examined their relationship with COMT genotype and memory functions. Previous studies have demonstrated an “inverted‐U‐shaped” function for the relationship between dopamine levels and cognitive performance (Cools & D'Esposito, 2011; Dickinson & Elvevag, 2009; Mattay, Goldberg, & Fera, 2003; Vijayraghavan, Wang, Birnbaum, Williams, & Arnsten, 2007), and a similar “inverted‐U‐shaped” relationship was also found for brain functional connectivity (FC) and participants with varying dopamine concentrations (Cole et al., 2013; Cools & D'Esposito, 2011; Tian et al., 2013). Thus, we hypothesized that the interaction of COMT Val^158^Met polymorphism and sex would modulate the association of FC strengths and memory performance. Specifically, we expected that male MM/MV carriers to have optimal dopamine level in the “inverted‐U‐shaped” function and the strongest FC strength that associated with higher memory performance.

## 2 MATERIALS AND METHODS

### 2.1 Participants

The study was approved by the Medical Research Ethics Committee of Nanjing Drum Tower Hospital. Two hundred young healthy Han Chinese participants were recruited from Nanjing, Jiangsu, China, by advertisements on the Internet. Exclusion of participants was performed via telephonic interview, based on the following criteria: (a) nonright handedness, (b) achromatopsia or hypochromatopsia, (c) smoking, (d) excessive drinking, (e) neurological and psychotic disorders or family history, (f) using drugs recently or drug abuse, and (g) pregnancy. All females were not in their menstrual period at the day of scanning and evaluation. Written informed consent was obtained from all participants. Financial compensation was provided after they completed all tests. One hundred and ninety‐six participants who met the criteria agreed to participate in the study. Nine participants were excluded due to failure of genotyping and one due to unsuccessful working memory test. Finally, data from 186 participants were included in subsequent analyses. All participants were divided into four groups based on their Val^158^Met genomic results (Met/Met and Met/Val (MM/MV) versus. Val/Val (VV)) and sex. The Met/Met and Met/Val participants were merged into one MM/MV group because of the low frequency of Met/Met homozygotes (4–5 times lower than Val/Val homozygotes) (Taylor, Züchner, & Payne, 2007; Xu, Qin, Liu, Jiang, & Yu, 2016b).

### 2.2 Genotyping

Genomic deoxyribonucleic acid (DNA) was extracted from white blood cells using a DNA extraction kit (BioTeke, Beijing, China) according to the manufacturer's instructions. COMT Val^158^Met (rs4680) genotypes of all participants were determined by Agena MassARRAY (Agena Bioscience). SNP primer was designed by Agena MassARRAY AssayDesigner3.1 Software. NanoDrop 2000 (Thermo Fisher, USA) was used to measure DNA concentrations. Data management and analyses were performed using the Agena MassARRAY TYPER4.0 Software. The rs4680 have a minor allele frequency (MAF) of> 5% in Chinese Han population.

### 2.3 Cognitive function assessment

#### 2.3.1 Episodic memory

The California Verbal Learning Test‐Second Edition (CVLT‐II) (Delis, 2000), which measures episodic verbal learning and memory abilities, was administered in a quiet room. The test contained two learning lists, A and B. List A comprised of 16 words drawn from four semantic categories, whereas list B comprised of 16 words drawn from two semantic categories of list A and two new semantic categories to create interference. First, participants were presented with list A over 5 learning trials and were asked to recall as many words as they can in any order (free recall) after each trial. Participants were then presented with list B. Free and cued recall of list A were tested immediately after list B (short‐delay), and again after 20 min (long‐delay). In cued recall, participants were prompted with the semantic category. Finally, participants performed a recognition task in which they indicated whether the presented item was a target word from list A or a distractor (total presented words, 48:16 from list A + 32 from list B and completely new words). Number of correct responses was recorded for the first time of list A (immediate recall), over 5 learning trials of list A (total recall), immediately after list B (short‐delay free and cued recall), and 20 min after list B (long‐delay free and cued recall). Recognition discriminability was calculated as d’ prime (i.e., numbers of hits minus false alarms).

#### 2.3.2 Working memory

Participants’ working memory capacity was measured using a n‐back behavioral task, performed outside scanner on a computer in a quiet room. E‐Prime 2.0 software (Psychology Software Tools, Pittsburgh, PA, USA) was used to present the visual stimuli, and participants’ button presses and response times were collected. Sixty blocks of 1‐back and 3‐back tasks were presented. Within each block, 60 letters were presented for 200 ms with an interstimulus time interval of 1,800 ms each. Participants pressed the right arrow button with their middle finger when the letter on the screen was identical to the one that was presented 1 (1‐back) or 3 (3‐back) letters earlier. Otherwise, they pressed the left arrow button with their index finger. They had to respond within a time period of 2,000 ms. Participants practiced the task before actual data collection. Proportion of correct responses and response times for 1‐back and 3‐back conditions were calculated, and working memory capacity was computed as the absolute value of proportion of correct responses for 3‐back minus 1‐back condition.

### 2.4 MRI acquisition

Participants underwent structural and RS‐fMRI scanning using the Philips 3T scanner (Ingenia, best, the Netherlands) with a 32‐channel phased‐array head coil. They were instructed to keep their eyes closed without falling asleep during scanning.

Structural 3D T1‐weighted images were collected using a brain volume (BRAVO) 3D‐FFE sequence (repetition time (TR)/echo time (TE) = 7.3/3.3 ms; inversion time = 450 ms; field of view (FOV) = 256 mm × 256 mm; matrix = 256 × 256; flip angle = 12°, slice thickness = 1 mm; no gap; 188 sagittal slices). RS‐fMRI images were collected using single‐shot echo‐planar imaging (TR/TE = 2000/30 ms; FOV = 220 × 220 mm; matrix = 64 × 64; flip angle = 90°; slice thickness = 3 mm; gap = 1 mm; 40 interleaved transverse slices; 180 time‐points excluding first 5 dummy scans for T1 equilibrium).

### 2.5 RS‐fMRI data preprocessing and analyses

RS‐fMRI data were preprocessed and analyzed using statistical parametric mapping (SPM12; Wellcome Trust Center for Neuroimaging) and FMRIB Software Library Multivariate Exploratory Linear Optimized Decomposition into Independent Components (FSL‐MELODIC; https://fsl.fmrib.ox.ac.uk/fsl/fslwiki/MELODIC). The quality of images was visualized to exclude scanner artifacts by an experienced radiologist. For each participant, volumes of the RS‐fMRI data were realigned to the first volume, unwarped, spatially normalized into Montreal Neurological Institute (MNI) space, resampled into 4 × 4 × 4 mm^3^ voxels, and smoothed using a 8mm full width half maximum (FWHM) Gaussian kernel. Framewise displacement (i.e., the averaged spatial displacement between two adjacent volumes) for the RS‐fMRI data was calculated for each participant, and those yielded an average framewise displacement of >0.2 mm were excluded in subsequent analyses.

The smoothed normalized RS‐fMRI data from all participants were then decomposed into 20 sets of spatiotemporal components using FSL‐MELODIC algorithm for independent component analysis (ICA) with a high‐pass filter of 125s to remove physiological noise and MCFLIRT motion correction to remove head motion ( Smith et al., 2009). Pearson's spatial cross‐correlations of the 20 components were calculated using ten well‐identified RSNs (Smith et al., 2009) to match the networks identified in our data and kept those components that yielded a significant spatial correlation (Pearson's *r* > 0.20). We found 16 components that were matched with the ten well‐identified RSN templates and 4 components that did not significantly correlate with any of the RSN template and likely due to artefactual effects (Reineberg, Andrews‐Hanna, Depue, Friedman, & Banich, 2015). Thus, 16 components were used to generate subject‐specific spatial maps and its associated time series using dual regression (Beckmann, Mackay, Filippini, & Smith, 2009). Specifically, for each participant, group‐averaged spatial maps were regressed (as spatial regressors in a multiple regression) into individual participant's 4D space‐time dataset. This resulted in a set of subject‐specific time series, one per group‐level spatial map. Next, the time series were regressed (as temporal regressors, again in a multiple regression) into the same 4D dataset to create a set of subject‐specific spatial FC maps of each network, one per group‐level spatial map (statistical threshold of Z> 2.3).

Finally, to investigate the COMT genotype and sex effects on RSN, we input the subject‐specific spatial FC maps of DMN, ECN, left FPN (LFPN), and right FPN (RFPN) into a second‐level analysis that modeled 4 RSN networks (DMN, ECN, LFPN, and RFPN) as a within‐subject variable and 4 groups (MM/MV males, MM/MV females, VV males, and VV females) as a between‐subject variable using SPM. Inferences were made at the second level to allow for random effects analysis and inferences at the population level (Penny & Holmes, 2007). At the random effects level, we tested for main effects of COMT and sex, and COMT genotype × sex interaction differences in each of the spatial maps. Unless otherwise specified, we report activations at family‐wise error (FWE) of *p < *.05 at the cluster level corrected for multiple comparisons across the whole brain (auxiliary threshold of *p < *.001 uncorrected). The search space for interaction effect was limited to the combination of the template of DMN, ECN LFPN, and RFPN as our search volume of interest (i.e., including 10,064 voxels) (Smith et al., 2009). For each participant, mean FC was extracted from the regions‐of‐interest (ROI) that showed significant effect of COMT genotype × sex and entered into an analysis of covariance to examine the influence of COMT genotype × sex interaction on associations between mean FC and working memory as well as episodic memory measures using IBM Statistical Package for the Social Sciences (SPSS) version 21.0. We also investigated the relationship between mean FC of the ROI and speculative dopamine level among groups by curve fit estimation in SPSS. Considering the “inverted‐U‐shaped” (i.e., nonlinear) model in dopamine neuromodulation, we used a quadratic model for the curve fit estimation. Taking into account of the effect of COMT genotype and estrogen on the dopamine level, female MM/MV carriers was hypothesized to have the highest dopamine concentration, whereas male VV carriers have the lowest.

### 2.6 Structural MRI (sMRI) preprocessing and analyses

The structural data were analyzed using SPM12. For each participant, the sMRI data were first segmented into gray matter (GM), white matter (WHM), and cerebrospinal fluid (CSF). The segmented images were then subsequently normalized to the averaged population templates generated from the complete image set using Diffeomorphic Anatomical Registration using Exponentiated Lie algebra (DARTEL) Tools (Ashburner, 2007), spatially normalized into MNI space and spatially smoothed using an 8mm FWHM Gaussian kernel. Total intracranial volume (TIV) for each participant was calculated as the sum of GM, WHM, and CSF volumes.

To investigate whether there are any main effects of COMT or sex, or COMT genotype × sex interaction on structural differences among the four groups, the smoothed GM images of each participant were entered into a full factorial 2‐way (genotype and sex) random effects ANOVA. TIV was included in the design matrix to account for intersubject variability due to head size. Unless otherwise specified, we reported significant differences at *p < .05* after the cluster level FWE corrected for multiple comparisons across the whole brain (auxillary threshold of *p < .001* uncorrected) within a specified search volume.

## 3 RESULTS

### 3.1 Demographic and genetic data

Table 1 shows the mean and standard deviation of age, education years, the scores of California Verbal Learning Test (CVLT), mean accuracy, mean response times, and capacity of n‐back tasks of the four groups based on COMT genotype and sex. The distribution of COMT Val^158^Met (8Met/Met (MM), 74Met/Val (MV), 104Val/Val (VV)) genotype was in Hardy–Weinberg equilibrium (*p*> .05). Given the low frequency of MM homozygotes (4–5 times lower than VV homozygotes), we merged MM and MV participants into one MM/MV group (Taylor et al., 2007; Xu, Qin, Liu, et al., 2016). Thus, in the final sample, there were 82 participants (27 males, 55 females) in the MM/VV group and 104 participants (31 males, 73 females) in the VV group. There was no main effect of COMT genotype, sex, and interaction of COMT genotype × sex (after correcting for multiple comparisons using false discovery rate ( Benjamini & Hochberg, 1995)) among four groups were found in any of behavioral measures (see Table 1).

**TABLE 1:** Demographic and neuropsychological scores of participants in COMT genotype‐defined and sex groups

	MM/MV		VV		*F* (3,182)	***p***
	Females (*N* = 55)	Males (*N* = 27)	Females (*N* = 73)	Males (*N* = 31)		
Age (years)	22.71 (1.66)	22.74 (2.07)	23.07 (2.10)	22.58 (1.61)	0.656	.580
Education (years)	16.42 (1.38)	16.56 (1.65)	16.89 (1.61)	16.52 (1.41)	1.146	.332
CVLT‐II						
List A first trial	7.49 (2.26)	6.93 (2.23)	7.27 (1.53)	6.77 (1.80)	1.147	.331
List A total trials 1–5	57.76 (8.91)	54.59 (11.75)	58.21 (8.11)	55.06 (9.54)	1.624	.185
Short‐delay free recall	13.40 (2.15)	12.74 (2.89)	13.42 (2.23)	12.35 (2.32)	2.035	.111
Short‐delay cued recall	13.78 (2.01)	13.22 (2.69)	13.73 (2.02)	12.61 (2.33)	2.430	.067
Long‐delay free recall	13.80 (2.15)	13.15 (2.90)	13.85 (1.92)	12.74 (2.16)	2.402	.069
Long‐delay cued recall	13.98 (1.96)	13.33 (2.86)	14.08 (1.82)	12.87 (2.08)	3.052	.030
Recognition discriminability	15.05 (1.28)	14.19 (2.56)	14.97 (1.89)	14.35 (1.45)	2.302	.079
*N*‐back						
1‐back accuracy	0.91 (0.04)	0.92 (0.05)	0.92 (0.04)	0.91 (0.04)	0.738	.531
1‐back response time (ms)	540.82 (95.27)	546.31 (85.33)	538.00 (93.61)	571.82 (108.36)	0.978	.405
3‐back accuracy	0.76 (0.17)	0.80 (0.15)	0.75 (0.15)	0.76 (0.12)	0.714	.545
3‐back response time (ms)	687.30 (169.86)	748.07 (174.87)	725.78 (175.39)	806.63 (198.88)	3.081	.029
Working memory capacity	0.15 (0.14)	0.13 (0.15)	0.18 (0.13)	0.17 (0.14)	0.049	.825

Note

Results are expressed as mean (*SD*). Variables were calculated by a 2‐way (genotype and sex) analysis of variance among four groups based on COMT genotype and sex to identify COMT × sex interaction effect. There is no significant difference among groups in terms of age and education years. CVLT and *n*‐back tests did not show significant difference among groups after false discovery rate correction.

COMT, catechol‐O‐methyltransferase; MM/MV, Met/Met and Met/Val; VV, Val/Val; CVLT‐II, California Verbal Learning Test‐Second Edition; *SD*, standard deviation.

### 3.2 Identification of the RSNs

**FIGURE 1:**
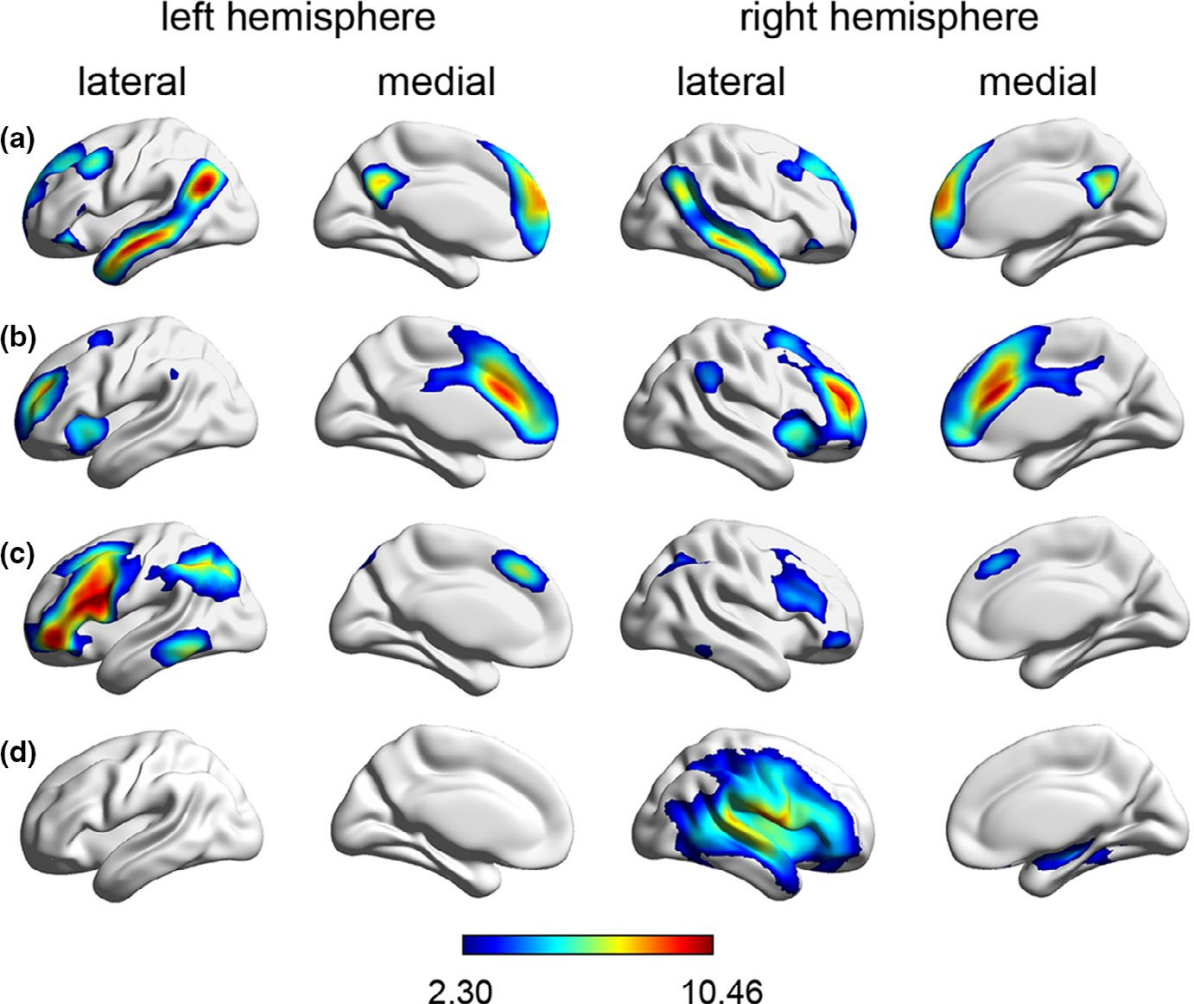
Cortical representation of the DMN (a), ECN (b), LFPN (c), and RFPN (d) identified by independent component analysis in our study group. Data are displayed on the lateral and medial surfaces of the left and right hemispheres of a brain surface map. The color scale represents the *t* values in each RSN. DMN, default mode network; ECN, executive control network; LFPN, left fronto‐parietal network; RFPN, right fronto‐parietal network

At the group level, FSL‐MELODIC ICA successfully identified DMN (*r* = 0.478), ECN (*r* = 0.691), LFPN (*r* = 0.660), and RFPN (*r* = 0.434) (see Figure 1). FSL‐MELODIC ICA also identified other RSNs, which overlapped with those described by Smith et al. (Smith et al., 2009). These included medial visual network (*r* = 0.743), auditory network (*r* = 0.640), and sensorimotor network (*r* = 0.735).

### 3.3 Influence of COMT Val^158^Met and sex on RSNs

**FIGURE 2:**
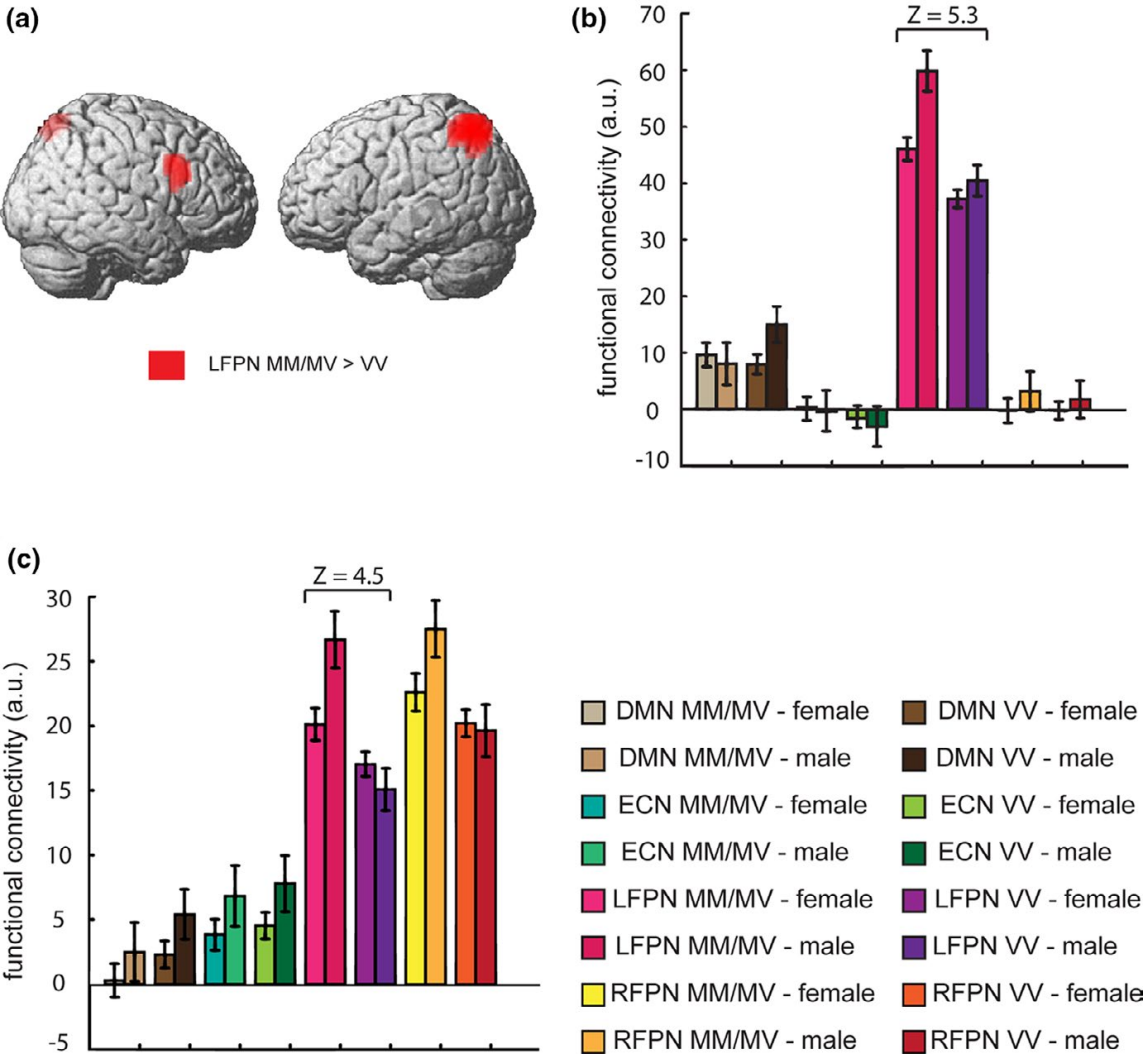
Effect of COMT Val^158^Met polymorphism on DMN, ECN, LFPN, and RFPN. (a) Increased functional connectivity for MM/MV relative to VV group in left superior parietal lobule and right inferior frontal gyrus (red) are rendered on a template brain. For illustration purposes, height threshold *p* < .001 uncorrected, >30 voxels. (b and c) Mean functional connectivity (across subjects’ mean ± *SEM*) for DMN, ECN, LFPN, and RFPN networks in MM/MV and VV groups at the peak MNI coordinates of (b) left superior parietal lobule (x = −42, y = −62, z = 56, Z = 5.3) and (c) right inferior frontal gyrus (x = 50, y = 18, z = 28, Z = 4.5) in LFPN. Error bars denote one *SEM*. COMT, catechol‐O‐methyltransferase; DMN, default mode network; ECN, executive control network; LFPN, left fronto‐parietal network; RFPN, right fronto‐parietal network; MM/MV, Met/Met and Met/Val; VV, Val/Val; *SEM*, standard error of mean

**FIGURE 3:**
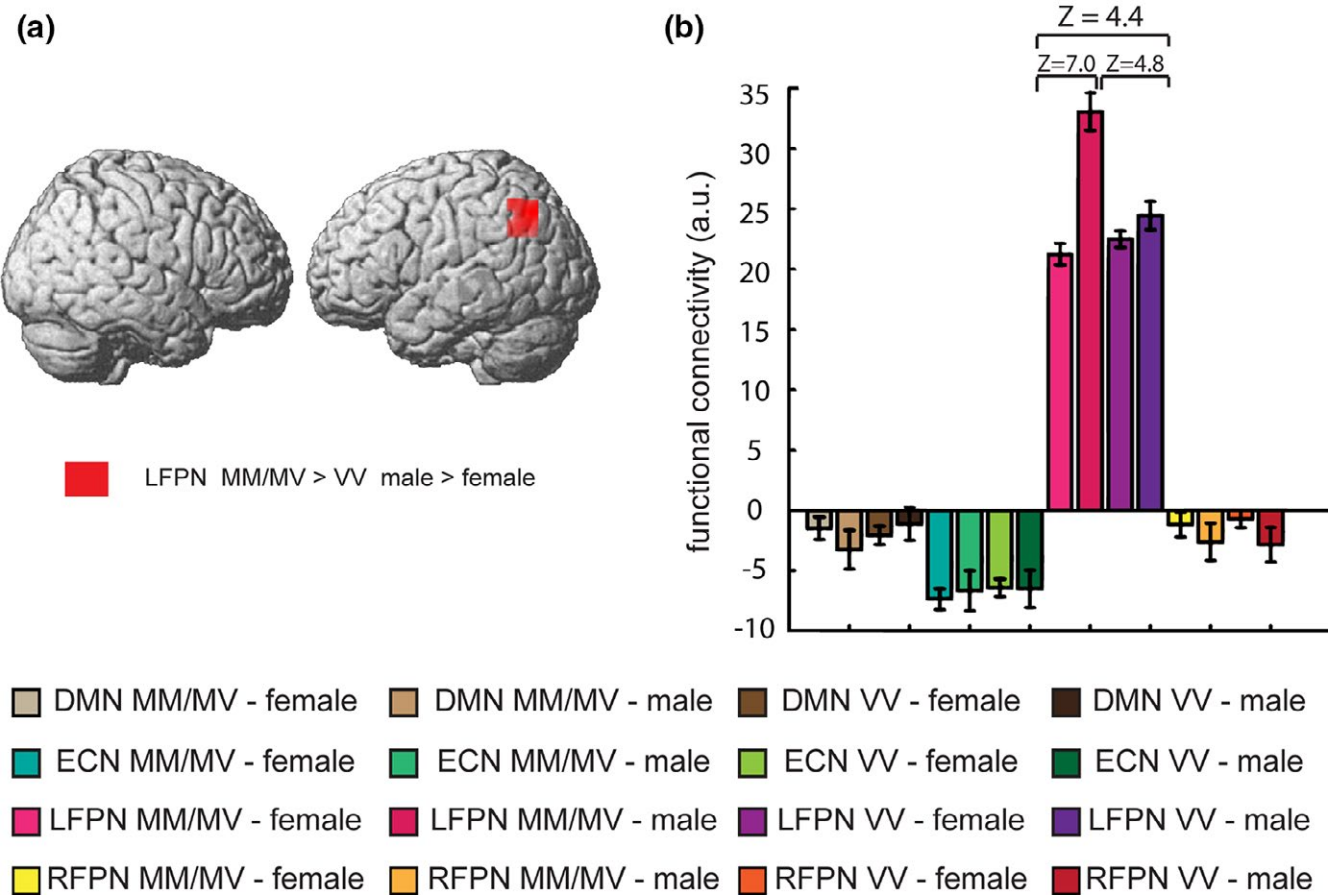
Effect of COMT Val^158^Met polymorphism and sex on DMN, ECN, LFPN, and RFPN. (a) Significant COMT × sex interaction on FC in left inferior parietal lobule (red) are rendered on a template brain. For illustration purposes, height threshold *p* < .001 uncorrected, >30 voxels. (b) Mean FC (across subjects’ mean ± *SEM*) for DMN, ECN, LFPN, and RFPN networks in four groups based on COMT genotype and sex at the peak MNI coordinates of left inferior parietal lobule (x = −30, y = −66, z = 40, Z = 4.4). Male MM/MV carriers showed significantly higher FC relative to male VV carriers (Z = 4.8) and female MM/MV carriers (Z = 7.0). Error bars denote one *SEM*. COMT, catechol‐O‐methyltransferase; DMN, default mode network; ECN, executive control network; LFPN, left fronto‐parietal network; RFPN, right fronto‐parietal network; MM/MV, Met/Met and Met/Val; VV, Val/Val; FC, functional connectivity; *SEM*, standard error of mean

Significant increased FC for MM/MV carriers relative to VV carriers was found in left superior parietal lobule and right inferior frontal gyrus in LFPN and not in other networks (see Table 2, Figure 2). Also, significant increased FC for males relative to females was found in each of the four networks (see Figure S1, Table S1). Furthermore, we found a significant COMT genotype × sex interaction on mean FC of left inferior parietal lobule (IPL) in LFPN spatial map within the search volume of interest (see Table 2, Figure 3a). Post hoc comparisons demonstrated increased mean FC for MM/MV group relative to VV group only in males (Z = 4.77, Figure 3b) and increased mean FC for males relative to females in COMT MM/MV group (Z = 7.03, Figure 3b). No COMT genotype × sex interaction on mean FC was found in DMN, ECN, and RFPN spatial maps. Also, we did not find any significant main effects on mean FC for VV relative to MM/MV group or for females relative to males in each of the four networks. Curve fit estimation in SPSS showed that the quadratic (inverted‐U‐shaped) model was found to be significantly fit (*R*‐square = 0.048, *p = *.011, see Figure S2).

**TABLE 2:** Effects of COMT Val^158^Met polymorphism and sex on LFPN

Contrast, brain networks and regions	MNI Coordinates			Cluster Size	Z Score (peak)	*FWE‐p*
	x	y	z			
LFPN						
Main effect of COMT						
MM/MV > VV						
L. superior parietal lobule	−42	−62	56	92	5.26	0.001
R. inferior frontal gyrus	50	18	28	41	4.54	0.031
VV > MM/MV	*N*.A.					
Main effect of Sex						
Males > Females						
L. inferior parietal lobule	−34	−70	44	1,107	7.53	<0.001
R. inferior parietal lobule	34	−70	36	116	4.51	<0.001
Females > Males	*N*.A.					
COMT × sex interaction effect						
MM/MV > VV, male > female						
L. inferior parietal lobule	−30	−66	40	60	4.35	0.009
MM/MV > VV, female > male	*N*.A.					

Note

The p value is corrected at peak level at *p < .05* for multiple comparisons using FWE method. The search space was limited to the combination of DMN, ECN, LFPN, and RFPN network (total, 10,064 voxels) as our search volume of interest.

COMT, catechol‐O‐methyltransferase; DMN, default mode network; ECN, executive control network; LFPN, left fronto‐parietal network; RFPN, right fronto‐parietal network; MM/MV, Met/Met and Met/Val; VV, Val/Val; R, right; L, left; FWE, family‐wise error.

### 3.4 Influence of COMT Val^158^Met and sex on gray matter volume

At the group level, a 2‐way (COMT genotype and sex) random effects ANOVA comparing GM volumes did not reveal any significant main effects of COMT or sex, nor effect of COMT genotype × sex interaction.

### 3.5 Examining the influence of COMT × sex interaction on associations between mean FC of LFPN and working and episodic memory measures

We examined whether the significant COMT genotype × sex interaction of mean FC of left IPL could be explained by differences in memory function. Table 3 summarizes the results of the univariate analysis of covariance. There was no significant main effects and interaction of COMT genotype, sex, and age on mean FC of left IPL. Significant association of mean FC for left IPL and CVLT first trial scores was found among four groups [*F*_(1, 174)_ = 7.28, *p = *.008]. Specifically, post hoc tests revealed a significant negative correlation between CVLT first trial scores and mean FC of left IPL in MM/MV group in males [*t*_(182)_ = −2.70, *p = *.008], thus suggesting that higher mean FC in left IPL was associated with worse episodic memory and, more specifically, immediate verbal memory in MM/MV male participants.

**TABLE 3:** Results for influence of memory function and the interaction with COMT × sex interaction on mean functional connectivity of left inferior parietal lobule

Effect	*F* (1,174)	*p*	Partial η^2^
Intercept	13.995	.000	0.085
COMT	0.370	.544	0.002
Sex	0.018	.893	0.000
Age	2.726	.101	0.018
CVLT List A first trial	5.047	.026*	0.033
CVLT List A total trials 1–5	3.029	.084	0.020
CVLT Short‐delay free recall	1.852	.176	0.012
CVLT Short‐delay cued recall	0.192	.662	0.001
CVLT Long‐delay free recall	1.629	.204	0.011
CVLT Long‐delay cued recall	4.706	.032*	0.030
Working memory capacity	1.409	.237	0.009
COMT × Sex	0.779	.379	0.005
COMT × age	0.597	.441	0.004
COMT × CVLT List A first trial	5.679	.018*	0.036
COMT × CVLT List A total trials 1–5	3.121	.079	0.020
COMT × CVLT Short‐delay free recall	0.152	.697	0.001
COMT × CVLT Short‐delay cued recall	0.772	.381	0.005
COMT × CVLT Long‐delay free recall	2.685	.103	0.018
COMT × CVLT Long‐delay cued recall	3.723	.056	0.024
COMT × Working memory capacity	0.861	.355	0.006
Sex × CVLT List A first trial	3.179	.077	0.021
Sex × CVLT List A total trials 1–5	6.408	.012*	0.041
Sex × CVLT Short‐delay free recall	5.278	.023*	0.034
Sex × CVLT Short‐delay cued recall	0.577	.449	0.004
Sex × CVLT Long‐delay free recall	3.634	.059	0.024
Sex × CVLT Long‐delay cued recall	2.371	.126	0.016
Sex × Working memory capacity	0.550	.459	0.004
COMT × Sex ×Age	0.569	.568	0.008
COMT × Sex ×CVLT List A first trial	7.283	.008*	0.046
COMT × Sex ×CVLT List A total trials 1–5	2.778	.098	0.018
COMT × Sex ×CVLT Short‐delay free recall	0.368	.545	0.002
COMT × Sex ×CVLT Short‐delay cued recall	0.073	.787	0.000
COMT × Sex ×CVLT Long‐delay free recall	0.298	.586	0.002
COMT × Sex ×CVLT Long‐delay cued recall	0.141	.708	0.001
COMT × Sex ×Working memory capacity	1.315	.253	0.009

Note

Data were analyzed using univariate analysis of covariance. *p < .05.

COMT, catechol‐O‐methyltransferase; CVLT, California Verbal Learning Test.

## 4 DISCUSSION

This study investigated the influence of COMT genotype and sex on intrinsic FC and memory performance in healthy young adults. Significant increased FC in male MM/MV carriers relative to male VV carriers and female MM/MV carriers was found in left IPL. Further, CVLT first trial performance was associated with the mean FC of left IPL in male MM/MV carriers. Collectively, our findings provide supporting evidence that male MM/MV carriers have increased functional coupling in LFPN that is associated with impaired performance in immediate verbal recall.

Previous studies that examined the effect of COMT genotype on cognition using resting‐state fMRI have typically focused on one type of cognition, for example, working memory (Zhang et al., 2015), executive function (Favaro, Clementi, & Manara, 2013), or decision making (Gao, Gong, & Liu, 2016). Yet, each of these resting‐state functional networks is not independent of each other and do not link to a single specific cognitive process, thus making it complicated to interpret the findings (Simon‐Vermot, Taylor, & Araque Caballero, 2018; Smith et al., 2009; Suri, Topiwala, & Filippini, 2017). Further, although memory is categorized into many sub‐types, each of these sub‐categories (e.g., working memory, short‐term memory, and episodic memory) shares many processing features (D'Esposito & Postle, 2015; Ranganath, Johnson, & D’Esposito, 2003). Thus, in the current study, instead of focusing on one aspect of memory function, we examined the effect of COMT genotype using different aspects of memory and RSNs that highly associated with cognitive functions.

In general, our findings are in accord with the hypothesis that MM/MV carriers have increased synaptic dopaminergic concentrations that result in higher intrinsic FC. Animal studies have shown that decreased COMT enzyme activity in Met relative to Val carriers leads to increased synaptic dopaminergic concentrations throughout the whole brain (Chen, Lipska, & Halim, 2004; Lotta et al., 1995). Further, neuropharmacological studies demonstrate that levodopa, which is used as a replacement for dopamine, optimizes the intrinsic FC in FPN (Cole, Oei, & Soeter, 2012; Simioni, Dagher, & Fellows, 2017). Collectively, these studies, together with our findings, suggest that higher intrinsic FC in MM/MV carriers is related to increased synaptic dopaminergic concentrations. Furthermore, human estrogen levels could also inhibit COMT gene transcription and down‐regulate COMT enzyme activity (Jiang et al., 2003; Xie et al., 1999) that affect intrinsic brain FC. Indeed, studies that identified sex differences on RSNs demonstrate higher FC for males relative to females (Biswal, Mennes, & Zuo, 2010; Filippi et al., 2013). Conversely, other studies show higher FC for females relative to males (Allen, Erhardt, & Damaraju, 2011; Bluhm, Osuch, & Lanius, 2008).

Interestingly, our findings reveal significant sex differences in mean FC of left IPL that is associated with CVLT first trial performance. Specifically, we found that male MM/MV carriers have increased functional coupling in LFPN that are associated with impaired performance in immediate verbal recall. IPL activity is correlated with many cognitive performance such as immediate recall score (Huo, Li, Wang, Zheng, & Li, 2018), episodic memory performance (He, Carmichael, & Fletcher, 2012), social cognition, and language (Bzdok et al., 2016). It is connected with other frontal and parietal regions and is considered as the node in FPN (Markett, Reuter, & Montag, 2014). The FPN encompasses lateral prefrontal cortex, anterior cingulate, inferior parietal lobules, and lateral cerebellum (Markett et al., 2014; Vincent, Kahn, Snyder, Raichle, & Buckner, 2008) and has been associated with working memory and executive control (Mohr, Wolfensteller, & Betzel, 2016; Sheffield, Repovs, & Harms, 2015). It is demonstrated that brain dopamine level strengthens FC in FPN, which positively correlates with working memory performance ( Simioni et al., 2017). In addition, previous meta‐analysis has demonstrated strong correlation between FPN and working memory (Rottschy, Langner, & Dogan, 2012). Collectively, these studies provide supporting evidence that synaptic dopamine concentration, which is influenced by COMT genotype and sex, plays an important role in the association between FPN intrinsic activities and immediate memory performance. Although CVLT is considered as a sensitive measure of episodic memory function, the meaning of different scores might vary among each other. Specifically, the immediate recall of first trial is related more to working memory, not episodic memory as compared to other CVLT scores (Wolk & Dickerson, 2011). In our study, different from our initial hypothesis, immediate verbal recall of CVLT first trial is negatively associated with spontaneous activity of fronto‐parietal working memory network in male MM/MV carriers, which suggests that the immediate verbal memory function is linked with enhanced inter‐regional desynchronization in resting state (Fjell, Sneve, & Grydeland, 2015). Analogous electrophysiological findings provide supporting evidence that demonstrate that strong desynchronization is associated with better verbal memory performance (Klimesch, Doppelmayr, Schimke, & Ripper, 1997) and thus is essential for large‐scale integration of human brain. RS‐fMRI results may provide similar information to understand the structure of human brain networks ( Laufs, Krakow, & Sterzer, 2003).

Additionally, our findings also provide support to the hypothesized model of “inverted‐U” (i.e., nonlinear) relationship between dopamine levels, regional brain response, and cognitive performance (Cole et al., 2013; Cools & D'Esposito, 2011; Dickinson & Elvevag, 2009; Mattay et al., 2003; Vijayraghavan et al., 2007). The “inverted‐U‐shaped” model suggests that an optimal level of dopamine is the most efficient, while suboptimal and supraoptimal dopamine level will impair brain activity and cognitive performance. In support to this, our findings demonstrate that mean FC of IPL in female MM/MV carriers is decreased relative to male MM/MV carriers, although the dopamine level of female MM/MV carriers should be higher considering Met and estrogen both down‐regulate COMT enzyme activity.

In accord to our findings, studies with schizophrenic patients provide additional supporting evidence, since variations of the COMT genotype have been implicated to have a strong link with memory performance of schizophrenia (Egan et al., 2001; Harrison & Weinberger, 2005; Twamley, Hua, & Burton, 2014). Deficits in immediate verbal memory are frequently reported in patients suffering from schizophrenia (Gold, Rehkemper, & Binks, 2000; Toulopoulou, Rabe‐Hesketh, King, Murray, & Morris, 2003). In addition, abnormalities in FPN are typically associated with schizophrenia (Xiang, Xu, & Wang, 2019), and task‐fMRI studies have demonstrated immediate memory modulation of reduced fronto‐parietal effective connectivity in schizophrenia (Nielsen et al., 2017).

We found no significant effects in GM volume within the search volume of interest, thus suggesting that the effects of COMT genotype and sex in healthy young participants manifest only at the functional level. Despite this, other studies have found mixed results: Some brain regions have either larger or smaller GM volume for Val relative to Met carriers (Cerasa et al., 2008; Taylor et al., 2007) and also a COMT × sex interaction effect on prefrontal GM volume ( Xu, Qin, & Li, 2016). The influence of COMT genotype and the interaction with sex on GM volume appears to be more prominent in populations who are at risk for psychosis relative to healthy volunteers (McIntosh, Baig, & Hall, 2007; Ohnishi, Hashimoto, & Mori, 2005).

Several caveats exist in our study. Contrary to previous studies that demonstrate that Met carriers have enhanced working memory capacity, our findings did not find any significant group difference in working memory capacity and association with brain function as measured using the n‐back task. Several studies have also found similar lack of association between COMT genotype and working memory in healthy young adults (Barnett, Scoriels, & Munafò, 2008; Wardle, de Wit, Penton‐Voak, Lewis, & Munafo, 2013). A possible explanation is that the n‐back task paradigm that we used in the current study is not sensitive enough to detect any significant group difference in young healthy participants as the performance of n‐back task is at ceiling (Buckert, Kudielka, Reuter, & Fiebach, 2012; Kane, Conway, Miura, & Colflesh, 2007). In addition, our sample size is comparatively small relative to other relevant genetic studies (Egan et al., 2001). Furthermore, although COMT enzyme activity and synaptic dopamine concentration in human brain are closely related to COMT genotype, we did not measure dopamine concentration in human brains directly, thus making the dopamine level hypothesis speculative. We also could not exclude confounding variable of fluid intelligence in our current study, as previous studies have found that COMT genotype influences on the neural circuitry supporting fluid intelligence (Bishop et al., 2008; Green, Kraemer, DeYoung, Fossella, & Gray, 2012). Still, whether participants keep their eyes open or closed might result in differences in brain intrinsic activities. Therefore, our results are limited in eyes‐closed condition and maybe not comparable with other similar studies with eyes open. Also, we did not record exact menstrual date of females while collecting the data. Future studies using larger sample size with a more direct manipulation of dopamine levels in human brain and greater task difficulty to measure working memory capacity and fluid intelligence will be required. The difference between eyes‐open versus. closed and the influence of menstrual date on behavior would also be interesting to explore in the future.

In conclusion, our study demonstrates that the COMT Val^158^Met polymorphism affects mean FC in parietal and frontal regions. Particularly, male MM/MV carriers, who are hypothesized to have higher dopaminergic concentrations, show higher FC in left IPL which is related to impaired immediate verbal recall performance, thus providing supporting evidence for the pathophysiology of schizophrenia.

## CONFLICT OF INTEREST

The authors report no biomedical financial interests or potential conflicts of interest.

## AUTHORS’ CONTRIBUTION

Sichu Wu: collected data, analyzed data and wrote the article; Neeraj Upadhyay and Jiaming Lu: Supported data analysis; Xueyan Jiang and Shumei Li: Supported statistical analysis; Qing Zhao: Supported article writing; Junxia Wang: collected data; Xue Liang: supported data collection; Xin Zhang and Bing Zhang: Project design and coordination.

## Supporting information

Supplementary Material

## Data Availability

Research data are not shared.
