# Supplementary material for: Interaction of Catechol‐O‐methyltransferase Val158Met polymorphism and sex influences association of parietal intrinsic functional connectivity and immediate verbal memory
Source: Brain Behav. 2020 Aug 8;10(10):e01784. doi: 10.1002/brb3.1784 (PMC7559624; doi:10.1002/brb3.1784)
Supplement: Supplementary file 1 — Supplementary Material [file BRB3-10-e01784-s001.docx]

Table S1 Effect of COMT Val^158^Met polymorphism and sex on DMN, ECN and RFPN.

| **Contrast, brain networks and regions** | **MNI Coordinates** | | | **Cluster Size** | **Z Score (peak)** | ***FWE- p*** |
| --- | --- | --- | --- | --- | --- | --- |
|  | **x** | **y** | **z** |  |  |  |
| **DMN** |  |  |  |  |  |  |
| **Main effect of COMT** | N.A. |  |  |  |  |  |
| **Main effect of Sex** |  |  |  |  |  |  |
| Males > Females |  |  |  |  |  |  |
| L. middle temporal gyrus | -46 | -54 | 12 | 141 | 6.77 | 0.000** |
| L. superior medial frontal gyrus | -10 | 54 | 32 | 218 | 6.11 | 0.000** |
| R. middle temporal gyrus | 58 | -54 | 20 | 197 | 6.05 | 0.000** |
| R. temporal pole middle temporal gyrus | 50 | 14 | -32 | 76 | 5.35 | 0.003* |
| L. middle temporal gyrus | -62 | -30 | -12 | 54 | 5.24 | 0.012* |
| L. precuneus | -6 | -54 | 16 | 280 | 5.18 | 0.000* |
| L. temporal pole superior temporal gyrus | -46 | 34 | -20 | 41 | 4.74 | 0.030* |
| L. superior frontal gyrus | -22 | 26 | 40 | 95 | 4.42 | 0.001* |
| Females > Males | N.A. |  |  |  |  |  |
| **COMT × sex interaction effect** | N.A. |  |  |  |  |  |
| **ECN** |  |  |  |  |  |  |
| **Main effect of COMT** | N.A. |  |  |  |  |  |
| **Main effect of Sex** |  |  |  |  |  |  |
| Males > Females |  |  |  |  |  |  |
| Medial, superior and inferior frontal gyrus | -30 | 18 | 0 | 1689 | 6.62 | 0.000** |
| Females > Males | N.A. |  |  |  |  |  |
| **COMT × sex interaction effect** | N.A. |  |  |  |  |  |
| **RFPN** |  |  |  |  |  |  |
| **Main effect of COMT** | N.A. |  |  |  |  |  |
| **Main effect of Sex** |  |  |  |  |  |  |
| Males > Females |  |  |  |  |  |  |
| R. inferior frontal gyrus opercular part | 54 | 10 | 20 | 102 | 4.56 | 0.001* |
| R. supramarginal gyrus | 54 | -26 | 32 | 59 | 4.13 | 0.009* |
| Females > Males | N.A. |  |  |  |  |  |
| **COMT × sex interaction effect** | N.A. |  |  |  |  |  |

The p value is corrected at peak level at *p < 0.05* for multiple comparisons using FWE method. The search space was limited to the combination of DMN, ECN, LFPN and RFPN network (total, 10064 voxels) as our search volume of interest.

COMT, catechol-O-methyltransferase; DMN, default mode network; ECN, executive control network; LFPN, left fronto-parietal network; RFPN, right fronto-parietal network; MM/MV, Met/Met and Met/Val; VV, Val/Val; R, right; L, left; FWE, family-wise error. *FWE *p < 0.05*. **FWE *p < 0.001*.


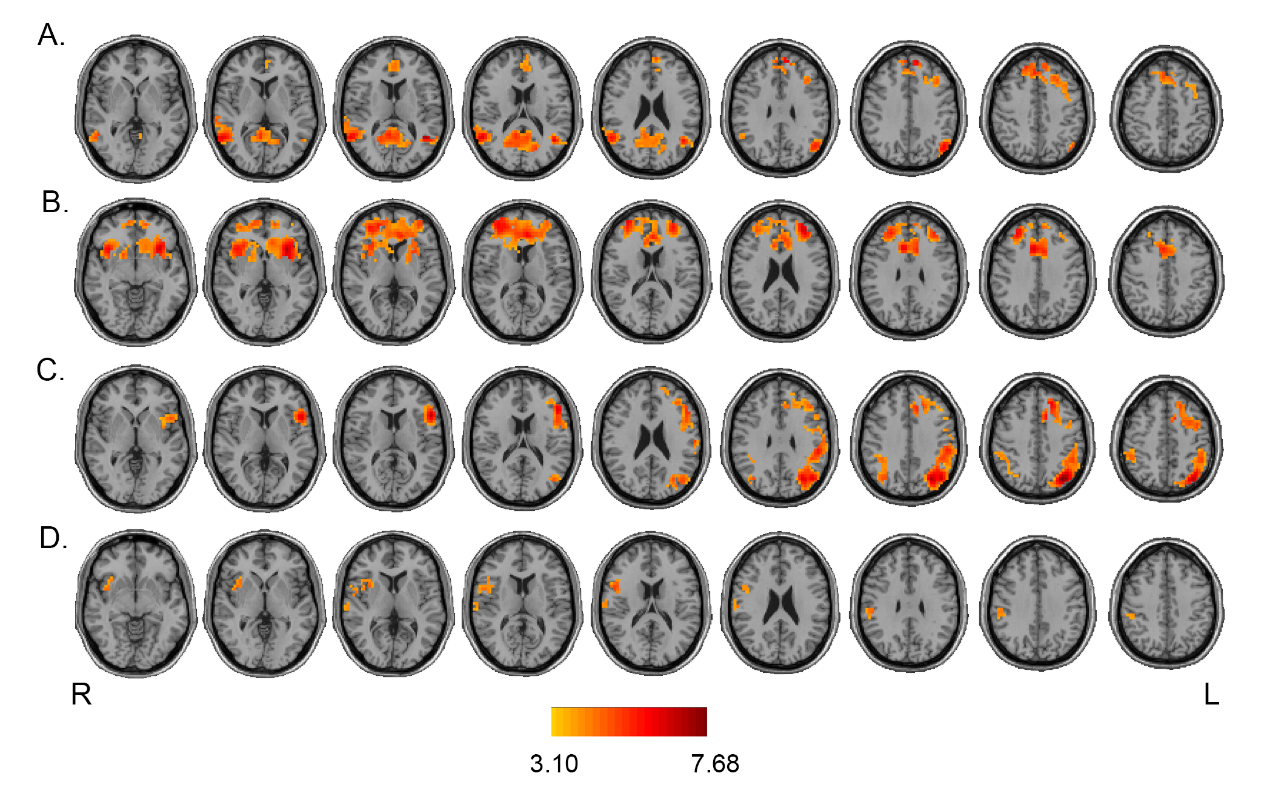


Figure S1 Effect of sex on DMN (A), ECN (B), LFPN (C) and RFPN (D). Increased functional connectivity for males relative to females was found in four networks. The p value is corrected cluster level at *p < 0.05* for multiple comparisons using FWE method. The search space was limited to the combination of DMN, ECN, LFPN and RFPN network (total, 10064 voxels) as our search volume of interest. No significant difference was found for females relative to males in these four networks.

DMN, default mode network; ECN, executive control network; LFPN, left fronto-parietal network; RFPN, right fronto-parietal network; FWE, family-wise error.


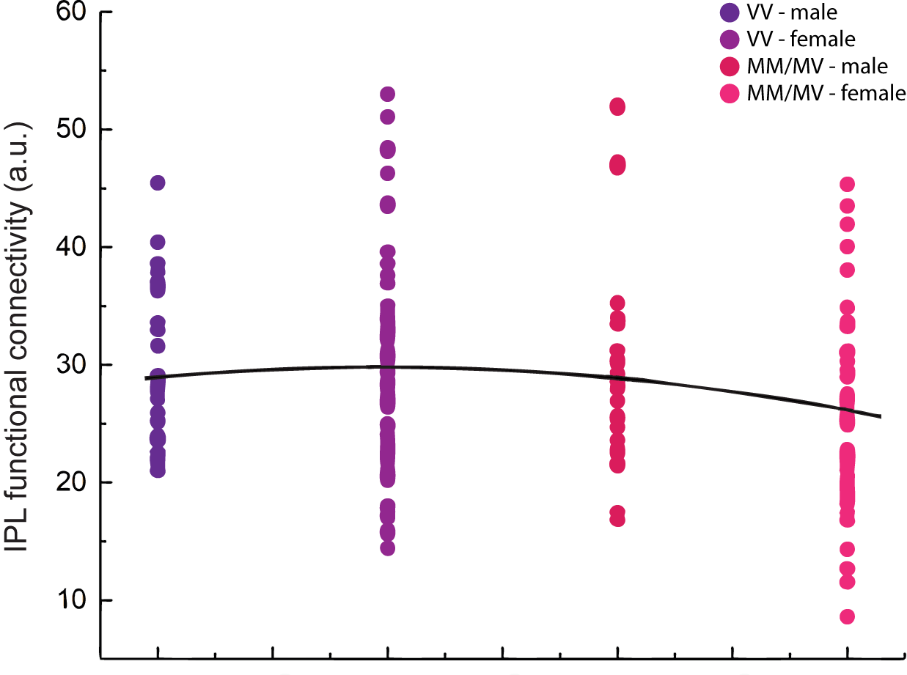


Figure S2 Results of quadratic curve fit estimations about the relationship between mean functional connectivity of left IPL and speculative dopamine level among groups.

IPL, inferior parietal lobule.
